# Techno-economic analysis and resilience enhancement of a hospital microgrid under grid outage scenarios

**DOI:** 10.1038/s41598-026-56638-8

**Published:** 2026-06-13

**Authors:** Michael Awad Eissa, Mohamed Mahmoud Samy, Mokhtar Said

**Affiliations:** 1 Electrical Engineering Department, Faculty of Engineering, Fayoum University, Fayoum, Egypt; 2 Electrical Engineering Department, Faculty of Engineering, Beni-Suef University, Beni-Suef, Egypt

**Keywords:** Microgrid, Renewable energy, Energy storage, Resilience, REopt, Energy science and technology, Engineering

## Abstract

Hospitals are critical infrastructures where power continuity is paramount. This study presents a techno-economic and resilience analysis of a grid-connected hybrid microgrid for a medium-sized hospital, comprising solar photovoltaics (PV), a battery energy storage system (BESS), and a diesel generator. Using a mixed-integer linear programming (MILP) model via NREL’s REopt^®^ platform, we optimized the system design to minimize the Net Present Cost (NPC) while ensuring an uninterrupted power supply to critical loads during grid outages. The analysis evaluated a wide range of outage scenarios, varying in duration (7–24 h), timing, season, and critical load level (50–100%). This study shows that a design of a microgrid for enhanced resilience is both technically and economically beneficial. The microgrid design with optimization achieves a net present cost savings of 14% for the financial optimization scenario and 9% to 14.2% for all the resilience-constrained scenarios compared to the business-as-usual case of 100% grid dependence. Most importantly, compared to the financial optimum rather than the business-as-usual case, the cost of including resilience constraints is a mere 0.4% to 2.4% of net present cost, showing that increased energy resilience can be delivered at a minimal cost. A key finding is that systems designed for summer outages yield higher savings due to greater solar availability, and a strategic deep-discharge protocol for the battery during emergencies is crucial for cost-effectiveness. This work provides an actionable framework for hospital administrators to enhance energy resilience without incurring a financial penalty, and in many cases, while realizing significant long-term cost savings.

## Introduction

### Background

A combination of factors, such as natural disasters caused by climate change, aging infrastructure, and the new threat of cyber-physical attacks, is increasingly posing a threat to the reliability of the electrical grid^1,2^. For the location of the present study, the U.S. Energy Information Administration indicates that the average electricity customer in Delaware faced 138 min of interruption in 2023, with a System Average Interruption Duration Index (SAIDI) of 138 min (excluding major event days). This data provides a measure of the expected vulnerability of the grid that would serve health care facilities in the Mid-Atlantic and so provides empirical justification for a resilience-focused microgrid study at this location. Wildfires, hurricanes, and extreme storms, which were previously perceived as rare occurrences, have been happening more often and more severely, causing extended power outages, imposing billions of dollars in expenses on the national economies, and affecting basic services^3,4^. Even though grid reliability has been the main objective in the past, resilience, which is the ability of a system to respond to a major disruption by preparing, withstanding, and quickly returning to normal, has become the primary goal of the modern power systems^5^.

The need for power resilience is especially intensive in hospitals. These facilities are complex ecosystems and will need a continuous high-quality source of electricity to perform many critical functions that are not just limited to direct support of life. Surgical equipment, diagnostic imaging machines (MRI, CT scanners), electronic health record systems, and HVAC systems that are required to maintain sterile settings and infection control all require a constant power supply^6^. Even the inconvenience caused by a short-term power outage may be disastrous and cause a chain reaction that endangers patient safety, distorts essential data, and might result in unhealthy results^7^. A 2017 power outage in one of the nursing homes in Hollywood, Florida, during Hurricane Irma, which killed several patients because of the lack of air conditioners, is one of the blunt reminders of the susceptibility of any healthcare facility^8^.

Diesel generators have been the most relied-upon tool for providing the backup power supply in hospitals. To a great extent, however, these systems are also subject to serious limitations, especially during long-term, extensive emergencies. They depend on expensive on-site fuel storage, and they are susceptible to interruptions of the refueling chain- a major flaw considering the roads can be impassable. Moreover, diesel generators produce negative greenhouse gases and particulate matter, are prone to mechanical breakdowns, and eventually, they are configured for short-term but not long-term outages^9^. A much more resilient, clean, and cost-effective solution towards greater hospital resilience is hybrid microgrids - localized power systems with their own production (like solar PV), energy storage, and controllable loads^10^.

Recently, the world has increasingly been experiencing energy challenges due to extreme weather, fluctuations in renewable energy production, and increased demand, causing concern with how to improve grid stability and reliability, beyond carbon reduction, particularly to essential facilities such as hospitals and data centers, susceptible to weather disruptions^11^. Traditional mitigation has traditionally been based on fossil fuel resources such as diesel generators as emergency backup, but this is unstable and expensive, as well as reliant on finite fuel sources, and emits^12^. Unlike renewable systems, especially solar PV systems with battery storage, which offer a cheaper yet more resilient alternative to critical infrastructure^13^.

### Literature survey

Recent research has shown that renewable energy systems can enhance resilience across several situations. As one example, when comparing Gdowski et al.^14^ to Rosales-Asensio et al., they disclosed that well-designed microgrids with energy storage systems offered excellent resilience in building power compared to ordinary backup generators and were cheaper and more reliable. Such systems will be viable only in terms of local factors, such as power demand and electricity tariffs, and the extra benefit such systems will have to business continuity in terms of outages is a consideration that cannot be ignored, even outside the purely quantified lifecycle cost analysis. Alexis Lagrange^15^ did the same with a hospital microgrid based on solar PV, batteries, and diesel generators. It was established in the study that solar PV would supply most of the power requirements of the hospital, with the batteries being utilized mostly during the peak hours to retain the value of the energy. The best periods of low-cost electricity were determined, and solar energy was scheduled as a priority on the high-demand days so that there would be less dependence on the diesel generation and increased resiliency^16^.

We should note the differences between the California hospital case study in ref^15^. and the current study. The previous work examined a large hospital in a location with high solar potential (GHI greater than 5.0 kWh/m²/day) and a large load profile. Our site in Delaware has a lower solar resource (avg. GHI of 4.27 kWh/m²/day), a more diverse climate with different winter heating needs, and a medium-sized hospital load (7.3 GWh/year). Apart from geographical differences, the key methodological difference is the analysis framework: while ref^15^. compared resilient designs to a no-grid baseline, we introduce an intermediate financial-only analysis that separates the structural savings of the microgrid from the incremental cost of resilience. This nested analysis allows for a more accurate economic analysis of resilience investments, addressing whether the cost of resilience is justified or is an additional cost to the hospital.

Recent developments have gone a step further than traditional microgrid components to explore the concept of all-inclusive, multi-carrier energy systems in the field of green buildings. Indicatively, Cicek et al.^17^ came up with a detailed operating model of a grid-interactive green building that incorporates renewable energy sources with a fuel cell, a hydrogen boiler, and plug-in electric vehicles (PEVs) and hydrogen electric vehicles (HEVs). Their work is the first to reveal how vehicle-to-grid (V2G) and vehicle-to-home (V2H) features, using battery and hydrogen storage in cars, provide essential backup in the case of grid failures, therefore, providing energy independence in buildings. Similarly^18^, used a similar multi-carrier, stochastic optimization model to apply to a hydrogen-based country house with its ability to optimize economically and continuously, even with extended outages, through utilizing its hydrogen infrastructure, including a hydrogen-powered fishing ship. Extending this multi-vector concept specifically to healthcare facilities, recent work has explored hydrogen-based energy management strategies for hospitals, demonstrating the potential of fuel cell systems to provide both primary power and long-duration backup during extended grid failures^19^. These studies indicate a radical advancement in the discipline, replacing crude PV-battery-diesel designs with complex, low-carbon, multi-fuel designs for residential and light commercial markets.

Despite these developments, gaps in the literature still remain. The majority of research is situation or place-specific and does not examine how renewable energy systems react to alternative levels of outage and load circumstances. Practical implementation of the more sophisticated, multi-vector energy principles to the specific environment of a hospital, with its intrinsic critical baseload and hierarchical service-priority structure, has not been studied. In addition, a number of articles are aimed at combining distributed generation (DG), energy storage systems (ESS), and renewable energy, such as wind and photovoltaics. Batteries constitute most electrochemical ESS currently, but the hybrid system and the electric vehicles (EVs) are rapidly increasing alternatives. The most common reliability assessment (RA) technique is the sequential Monte Carlo simulation, and emerging techniques like artificial intelligence (AI) are gaining momentum. This is especially true among the development of advanced metaheuristic optimization algorithms for power systems. The growing complexity of these systems has led to a growing interest in artificial intelligence and machine learning techniques for demand forecasting and predictive energy management, with recent research that shows how AI-based reserve management can enhance microgrid reliability during islanded operation^20,21^.

Concurrently, advanced metaheuristic optimization algorithms have demonstrated effectiveness in solving complex power system problems, including economic load dispatch and optimal generator allocation^22–24^. However, these algorithmic developments represent a separate research trajectory from the MILP-based REopt optimization employed in the present study^25–27^.

The current literature review presents an evident movement in the subject. Preliminary research studies have proved the techno-economic feasibility of photovoltaic-battery-diesel microgrids, which provide a strong foundation to carry out further research. Recent state-of-the-art studies such as^17,18^ have extended the discussion to include hydrogen as a key energy carrier and demonstrated the usefulness of mobile storage in stabilizing the grid in residential green buildings. At the same time, models of community-level optimization, including the one that is suggested in^28^, are starting to consider the complexity of shared energy resources. However, there is still a significant gap in terms of the implementation of these progressive ideas in the context of a unique and essential healthcare facilities environment, which has a radically different profile of energy needs. The current research attempts to fill this gap by implementing an advanced techno-economic and resilience model to fit the hospital environment, which is why the explicit focus of the study is on the unique operational demands and resilience requirements of the healthcare industry.

The emergence of adaptable protection systems, healthy communication networks, and consistent power-electronic subsystems is a daunting endeavor, especially when it comes to heterogeneous microgrid topologies, advanced forecasting techniques, and the widespread impact of information and communications technology on microgrid reliability. These interdependencies give rise to the need for continued research in future research studies^29^.

More recent novel projects have presented the concept of resilient microgrid architectures based on a three-stage plan to first optimize component sizes, then reduce unserved demand during disruptive events, and finally add systemic redesign to enhance overall resilience^30^. These models introduce systematic approaches to improve the stability and functional effectiveness of microgrids during stressful infrastructure conditions, as it was demonstrated in the Roosevelt Village project that design optimization has a significant beneficial effect on the level of performance indicators. Combining renewable generation resources with developed energy storage systems has yielded measurable benefits in three key areas: reduction of operating energy costs, carbon reduction, and increased power reliability to critical loads during grid disturbances. Additionally, it shows that strategic energy savings can be effectively used to develop renewable integration and thus help the establishment of sustainable and economical housing solutions^31^.

The techno-economic assessment of microgrids, particularly for off-grid communities and military bases, is well-established^32^^33^,. These foundational studies have consistently demonstrated the economic and operational feasibility of optimizing the size and dispatch of Distributed Energy Resources (DERs). Complementary to this, recent research has advanced the application of novel optimization algorithms for solving complex power system problems, such as the Economic Load Dispatch (ELD) and the optimum allocation of generator units. For instance, studies by Ismaeel et al. have demonstrated the effectiveness of metaheuristic techniques like the Osprey Optimization Algorithm and Snow Ablation Optimization in achieving efficient and cost-effective solutions for these fundamental problems^34,35^. Further work by Said et al. has shown the promise of the Walrus Optimizer in tackling the ELD problem, highlighting the continuous evolution of optimization tools available for system planning^36^.

In addition to the optimization of microgrids, the empirical reliability testing of the subsystems and the emergent approaches have been tested and have shown that even though batteries are prevalent in energy storage, hybrid systems and electric vehicles are alternatives that can be adopted. Furthermore, although traditional methods of evaluation are still commonplace, AI-focused approaches are gaining more and more recognition; however, obstacles in the creation of more flexible protection systems, resilient communication systems, and reliable power electronics still persist, thus highlighting the need to conduct further research on different microgrid designs, powerful forecasting algorithms, and the use of ICT in the context of increased reliability^29^.

Sustainable backups are biofuels, but the renewable technologies, such as solar photovoltaic systems, are getting increasingly resilient and cost-effective and have strategic advantages such as minimized fuel reliance and a significant cost reduction^37,38^. Renewable systems can sustain critical loads during power outages, especially in large office buildings where data servers can be found^38–40^. Table 1 is the summary of the recent feasibility tests on the components of microgrids, analysis programs, and outage behavior^41^.

Although these developments have been made, the resilience of infrastructure has received much scholarship, such as military installations^35^ or airports^42^. The recent study, which expands upon the earlier work by the author^43^, adds weight to the economic feasibility of resilience-based microgrid designs at hospital locations. However, this was only analyzed at one critical level of load, which was 50% critical. The specific features of the energy demand of the particular hospital, and in particular the non-variable, 24-hour baseload needed to support life-threatening services, have received relatively little inspection. The present investigation aims to eliminate this particular gap through the introduction of a comprehensive techno-economic and resilience framework directly into the hospital context, thus providing a custom-made study on one of the most important infrastructures within society.

Despite the above progress, there remain two key gaps in the hospital microgrid literature. First, previous hospital-specific studies^15^ have not defined a base case based on pure economic optimization against which the incremental cost of resilience may be determined; this prevents them from determining whether resilience is achieved at a cost premium or discount compared to a purely economic design. Second, the effects of critical load size, outage duration, and seasonal timing on optimal size and dispatch have not yet been explored through a comprehensive sensitivity analysis across critical load levels, outage lengths, and seasonality. The current research fills these two gaps by providing a hierarchical approach for economic comparisons and a sensitivity matrix of 50%, 70%, and 100% critical loads under 7-hour and 24-hour outages across all seasons.

### Aim and contributions

This paper, therefore, uses the REopt^®^ software to model and optimize a microgrid of a hospital in Wilmington, DE, thus filling in the salient gaps in the available literature. Based on previous studies, we consider the ability of critical facilities to achieve a reliable electric power supply in the event of grid disconnection and, in particular, the operational robustness of a grid-connected microgrid, which includes photovoltaic solar arrays, battery storage, and a diesel generator that has the capacity to maintain the critical infrastructure of a hospital through prolonged grid outage at a minimum overall operating expense.

This study’s key contributions to previous hospital microgrid studies are:

i. Estimation of incremental cost of resilience via a financial-only baseline. In contrast to past REopt hospital case studies that compared resilient designs with a business-as-usual (BAU) grid-only scenario, we include an intermediate financial-only scenario. This scenario provides a direct comparison and shows that the design cost savings of a PV-plus-battery microgrid are so large (14% lower NPC vs. BAU) that the incremental cost of including resilience constraints is 0.4–2.4% on the net present cost. This insight gives hospital administrators a key value metric: resilience at nearly zero incremental cost.
ii. A sensitivity matrix of interacting outage variables. We vary the duration of the outage (7–24 h), the level of critical load (50%, 70%, and 100%), the season in which the outage begins (winter, spring, summer, and autumn), and the time of day of the outage onset. This four-dimensional approach reveals how optimal system size, battery capacity, and diesel generator use vary across these variables - something that has not been done in previous hospital energy system studies, which typically considered just one or two outage durations at a single critical load level.
iii. Cost avoidance through operational deep-discharge of the emergency battery. We show that allowing the battery state of charge to drop to 0% only during grid outages (with a 20% minimum state of charge otherwise) prevents battery oversizing while ensuring critical load coverage. This operational insight, together with the lifecycle cost analysis, offers an explicit design rule for resilience-driven battery sizing in critical infrastructure.

### Paper organization

The structure of this paper is as follows: Sect. 2 presents the microgrid architecture proposed and its main elements. Section 3 elaborates on the methodology and approach to analysis. The results of the simulation, comparative analysis, and discussion are discussed in Sect. 4. Lastly, Sect. 5 completes the paper with the conclusion about important findings and research directions in the future.

**Table 1:** Comparative summary of recent microgrid feasibility studies:.

Study	Microgrid composition	Optimization platform	Load type	Lost load values	Outage scenarios	Key contribution
^44^	PV/Wind/BESS/DG	HOMER	Industrial parks (Ethiopia)	Not Modeled^1^	1.5–2.5 h (random)	Grid integration reliability
^45^	PV/BESS/DG	HOMER Pro	Civic center (Tanzania)	Not Modeled	1 h–5 days (scheduled)	Blackout impact on hybrid system viability
^46^	PV/BESS	HOMER	Residential (Pakistan)	Not Modeled	6 h (daily)	Grid-tied PV feasibility under unreliable grid
^47^	PV/BESS	HOMER	Household (Iraq)	Not Modeled	2 h (random)	Islanded vs. grid-connected operation
^48^	PV/BESS/DG	HOMER	Rural community	Not Modeled	9 h (daily)	Power management in remote areas
^49^	PV/BESS/DG	HOMER	Glass plant (India)	Not Modeled	1 h (random)	Industrial site reliability
^50^	PV/BESS/Gas gen	HOMER	Healthcare clinic (Iran)	Not Modeled	48 h (annual)	Traditional vs. renewable backup
^14^	PV/BESS/DG	REopt	Workplace (California)	Not Modeled	1–336 h (random)	Resilience cost quantification
^51^	PV/BESS	REopt	Mixed-use (ICE tool profiles)	ICE tool values	35 h (annual)	VoLL impact on optimal sizing
^52^	PV/BESS/DG	REopt	Workplace/School/Hotel (US)	ICE tool values	2 h (annual)	Multi-building resilience valuation
^15^	PV/BESS/DG	REopt	Hospital (California)	$100/kWh	8/16/24 hr	First hospital-specific resilience optimization
^53^	PV/BESS/DG	REopt	Medium business (California)	Not Modeled	1–9 days (random)	Long-duration outage planning
^15^ ^[,42^	PV/BESS/DG	REopt	Medical center (New York)	$100/kWh	1.8 h (annual)	Urban healthcare resilience
^54^	BESS/PV/NG/wind	HOMER Grid	Hospital/Hotel/Office (US)	$100/kWh	72 h	hospital resilience 40%more than hotel
^17^	PV/Wind/FC/H₂ Tank/PEV/HEV	MILP	Residential Green building	Cost minimization	Grid outage, H₂	PEV + HEV V2G/V2H for resilience
^18^	PV/Wind/FC/H₂ Tank/H₂ Vehicles/Boat	MILP	Residential Country house	Cost minimization	Grid outage daily	H₂-based country house study
^28^	PV/ESS/FCEV (V2G)	MILP	Residential community	Implicit resiliency for sensitive loads	Abnormal conditions of the grid	model with H₂ and V2G
This study	PV/BESS/DG	REopt	Hospital	$100/kWh	7–24 h	hospital resilience optimization

^1^Note: ‘Not Modeled’ means that the original study did not use a value of lost load in its optimization model; ‘Not Reported’ denotes that the study may have used a lost load, but it did not clearly state what value was applied. ICE tool values Interruption Cost Estimator of Lawrence Berkeley National Laboratory.

## System description and modeling

**Fig. 1:**
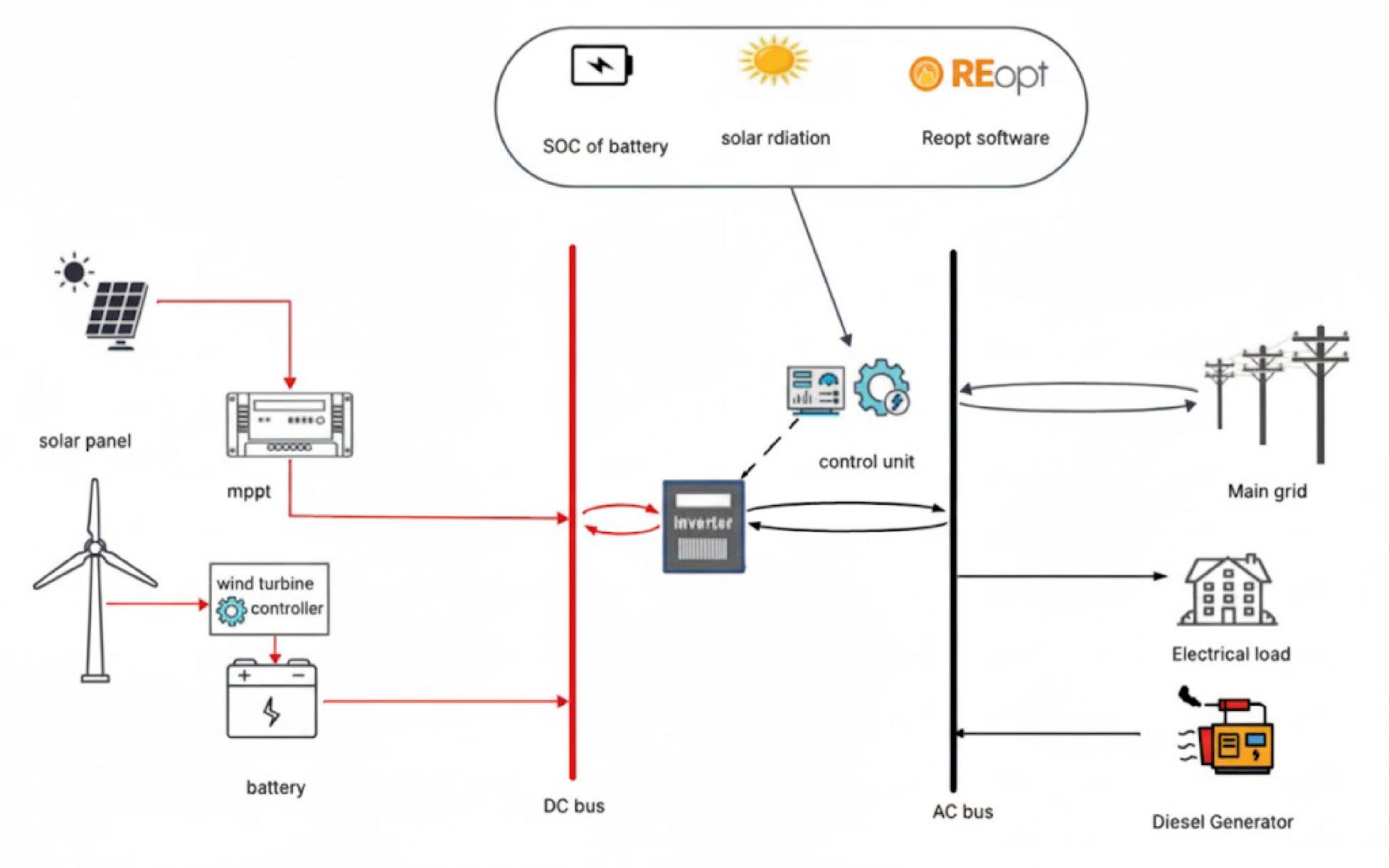
Microgrid architecture for the simulations.

As shown in Fig. 1, the proposed resilient hospital microgrid follows the architectural design that will be able to work Alongside these advances with the main utility grid and switch to the islanded mode during outages. The fundamental elements include a solar photovoltaic (PV) array, a battery energy storage unit (BSS), and a normal diesel generator (DG). A microgrid controller is in charge of these assets, optimizing power flow to serve the tiers of needs of the hospital, which include high-critical, low-critical, and noncritical, to ensure that the most critical services are made available under all circumstances.

### Photovoltaic system

The grid-connected photovoltaic (PV) system viability was evaluated through the analysis of site-specific solar resources and modern techno-economic variables. The measurement used local hourly Global Horizontal Irradiance (GHI) measurements to generate an accurate system energy production model.

Figure 3 below shows that the yearly solar resource profile of the region has sharp diurnal and seasonal variations. The maximum values of irradiance are always around noon in the day, and the highest values are during the spring and summer seasons, hence validating that there is a high potential for solar power at the study site^55^.

In this study, a fixed tilt and south-facing rooftop PV station was modeled. It was designed with a total system loss of 14% due to dust deposition, partial shading, diode losses, contact resistance, system aging, and light-induced degradation^46^. This 14% derating factor corresponds to the default system loss assumptions in the System Advisor Model (SAM) of NREL of rooftop photovoltaic systems and industry practice when doing preliminary feasibility studies. The factor naturally explains combined losses due to soiling (2–5%), wiring and mismatch (2–3%), nameplate rating tolerance (1%), and availability (2%). Nonetheless, the nature of hospital rooftops poses special shading issues since they include large heating, ventilation, and air conditioning (HVAC) units, exhaust stacks, and helipad constructions that are not typical of commercial buildings. To design a final project, it is highly suggested that a detailed on-site shading analysis with LiDAR or similar three-dimensional modeling be conducted to determine whether site-specific obstructions may result in higher overall system losses than the 14% baseline assumption. Furthermore, the REopt model includes a PV degradation rate, which is an annual rate, that is used to represent the gradual loss in efficiency throughout the 25-year analysis period.

The financial calculations were on the basis of a capital expenditure of 1600/kW-DC and annual operating cost of 16/kW-DC (detailed input parameters are provided in Appendix A). These data, which included resource metrics and engineering parameters, were directly introduced into a techno-economic model and made it possible to quantify the system energy output, evaluate the economic viability, and estimate the ability of the system to compensate for the carbon emissions.

1 $$\:{P}_{pv}\left(t\right)=\mathrm{G}\left(\mathrm{t}\right).\mathrm{A}.{{\upeta\:}}_{pv}.\left(1-losses\right)\:$$

Where:

- $\:{P}_{pv}\left(t\right)$ refers to the power generated by photovoltaics in kilowatts.
- *G(t)*: is the irradiance of the sun in kilowatts per square metre.
- *A*: is the total area of the panel, taken in square meters.
- $\:{{\upeta\:}}_{pv}$: is the efficiency of the photovoltaic system is normally measured between 15% and 20%.
- *losses*: Total losses due to dust, shading, aging, etc. (e.g., 14%)

### Battery system configuration

Battery storage set-up, as well as the policies that correspond to it, is highly varied under certain conditions of a particular case^56^. In the current study, the minimum state-of-charge (SOC) during normal grid-connected operation was set to 20%. This is the industry standard energy storage system of lithium-ion batteries, and it indicates the specifications of manufacturers to maintain the cycle life. Regularly working lithium-ion cells below 20% SOC speeds up capacity degradation by processes such as anode copper dissolution and solid electrolyte interphase (SEI) growth^57^. Maintaining this minimum when cycling normally would mean that the battery would last its design lifetime of 10 years before it needs to be replaced (according to Appendix A). This normal-operation limit should be contrasted with the emergency deep-discharge procedure used in the case of a grid outage, which is an explicitly programmed exception in Sect. 4.2.2.2 and which allows the SOC to go to 0% only during infrequent emergency situations in order to optimize the use of stored energy^58^.

The battery system costs include $420/kWh for energy capacity and $968/kW for power capacity, with annual O&M at 2.5% of capital cost and replacement scheduled in year 10 [Appendix A].

• Charging mode: On the energy absorption side, the state of charge (SOC) evolves by the relation below:

2 $$\:{B}_{soc}\left(t\right)={B}_{soc}\left(t-1\right)+{P}_{ch}\left(t\right).{{\upeta\:}}_{ch}$$

• Discharging mode: The loss of the SOC is expressed in the delivery of energy:

3 $$\:{B}_{soc}\left(t\right)={B}_{soc}\left(t-1\right)-\frac{{P}_{dis}\left(t\right)}{{{\upeta\:}}_{dis}}$$

Where:

$\:{P}_{ch}\left(t\right):\:\:$the charging power at step t of time.

$\:{P}_{dis}\left(t\right)$ : the power that is discharging at time step t.

$\:{B}_{soc}\left(t\right)$ : the state of charge of the battery.

$\:{{\upeta\:}}_{dis}$ : efficiency of the battery to discharge.

$\:{{\upeta\:}}_{ch}$ : the efficiency of the battery charging.

### Diesel generator

A diesel generator is included in the microgrid architecture as a key component for enhancing resilience, providing a dispatchable power source to sustain the critical load during a grid outage. The generator works in harmony with the photovoltaic array and the Battery Energy Storage System (BESS) in order to ensure the power supply is not interrupted.

For this analysis, the generator is modeled as an emergency backup unit, primarily intended to operate only when the grid is unavailable. The optimization model dispatches its operation to meet any portion of the critical load that cannot be served by the available solar generation or the stored energy from the BSS. The model assumes the generator can run in parallel with the other distributed energy resources while the system is islanded.

The economic analysis for the diesel generator was based on a representative capital cost for an emergency backup unit. Consistent with the default parameters in the REopt model, an installed capital cost of $500 per kW was assumed for a generator that operates only during outage conditions^59^[see Appendix A for complete generator parameters, including fuel cost and efficiency]. The diesel generator was used to act as a backup in case of a shutdown. It has a linear proportion when it comes to its consumption rate of fuel, and the output of power, which can be given as^60^.

4 $$\:F={\mathrm{F}}_{0}.{\mathrm{Y}}_{gen}+{\mathrm{F}}_{1}.{\mathrm{P}}_{gen}$$

Where:

F: fuel consumption rate (L/hr).

#### $\:{\mathrm{F}}_{0}$

no-load fuel consumption ratio(L/hr/kW-rated).

#### $\:{\mathrm{Y}}_{gen}$

Rated generator capacity (kW).

#### $\:{\mathrm{F}}_{1}$

Marginal fuel consumption coefficient (L/hr/kW-output).

#### $\:{\mathrm{P}}_{gen}$

Electrical Power output of the DG (kW).

Model Limitations. The linear fuel consumption model of Eq. (4) makes a reasonable first-order approximation of steady-state operation of a generator but fails to reflect a number of real-world inefficiencies, which are of interest to the application of hospital backup power. Namely, the model fails to capture (i) cold-start fuel penalties, in which electric generators that have been idle burn more fuel during the initial warm-up process before they stabilize at operating temperature. (ii) transient load-following inefficiencies, whereby rapid changes in hospital electric demand (e.g., the simultaneous start-up of multiple large medical devices) cause temporary rich-burn conditions, which burn more. Consequently, the fuel consumption and emissions recorded in this research are to be considered as a lower-bound estimate. An area of future work is the recommended dynamic diesel generator model that accounts for cold start and transient behavior and is necessary to be included in detailed engineering design.

### Wind turbine system

In addition to solar resources, the microgrid design considers a wind turbine to supplement on-site generation, diversify the renewable energy portfolio, and enhance system resilience. The wind turbine converts kinetic energy from the wind into electrical power that can directly serve the hospital’s load or charge the Battery Energy Storage System (BSS).

The performance of the wind turbine is modeled using location-specific wind resource data to generate an hourly power production profile. The optimization model then determines the cost-optimal size of the wind turbine, considering its potential to reduce grid electricity purchases and its contribution during potential outage scenarios. The dispatch of the wind turbine is prioritized to serve the load whenever the wind resource is available.

The study site has a yearly average wind speed of 5.35 m/s at 50 m above ground (see Table 2), which is the lower end of what is considered economically feasible for small and medium wind turbines. The REopt model includes the option of using wind power and endogenously optimizes the capacity based on hourly wind resource data and the cost of wind power. As shown in the results in Sect. 4, the model generally selects very low (or zero) wind capacity across most scenarios, suggesting that, given current capital costs and the site-specific wind resource, wind power is not economically viable for this case study. Thus, while the use of wind should be considered a test of the model’s capability, it should not be considered a design recommendation.

The economic analysis for the wind system was based on a fully installed capital cost of $4,760 per kW and an annual operations and maintenance (O&M) cost of $42 per kW. These cost parameters are used to evaluate the financial viability and role of the wind turbine within the overall least-cost microgrid configuration^61^[Appendix A].

The energy of the wind and the efficiency of the turbine are major factors that control the power output of the wind turbine. Most of the time, this relationship can be expressed in the following equation^62^:

5 $$\:{P}_{wt}\left(t\right)={\frac{1}{2}C}_{p}\rho\:A{V}^{3}$$

Where:

$\:{P}_{wt9}$ is the wind turbine power output at time t.

$\:{C}_{p}$ is the power coefficient, representing the turbine’s efficiency.

$\:\rho\:$ is the air density (in kg/m³).

A is the swept area of the rotor.

V is the wind speed.

### Cost model

The Capital Recovery Factor (CRF) determines the costs of the present values in the form of annual costs, with consideration of the time value of money^63^. It is calculated as:

6 $$\:CRF(r,m)=\frac{{r(1+r)}^{m}}{{\:\:\:\:\:\:\:\:\:\:(1+r)}^{m}-1}$$

Where:

r: real interest rate.

m: project life time.

y: equipment lifetime.

**Annualized Costs**.

- Capital cost:$\:\:{\mathrm{C}}_{cap}$∗ CRF(r, m).
- Replacement cost: $\:{\mathrm{C}}_{rep}$∗ CRF(r, m) ∗ $\:\frac{1}{{(1+r)}^{y}}$.
- The total annual expenditure is the amount of annualized capital expenditure, annual operation and maintenance expenses, annual replacement cost, and annual salvage value.

Levelized Cost of Electricity:

Levelized Cost of Electricity (LCOE) is the average cost of one unit of electricity produced in the entire life of the project that includes all capital, operations, and replacement costs. It is calculated as^64^:

7 $$\:LCOE=\frac{\mathrm{A}\mathrm{C}\mathrm{S}}{Total\:electrical\:load\:served}$$

Where:

ACS: Annualized Cost of the System, which is similar to the Total Annual Cost (TAC).

Total electrical load: the amount of electricity (in kWh) supplied to the system in a year.

The LCOE can be considered an important parameter used to compare the economic attractiveness of different energy systems^65^.

## Proposed tools

**The REopt** ^®^

The model tool used in this study is the REopt^®^^59^, and it was used to analyze the integration of renewable energy and to design storage systems with the dual objective of reducing spending on energy while enhancing microgrid resilience^66–68^.

**Fig. 2:**
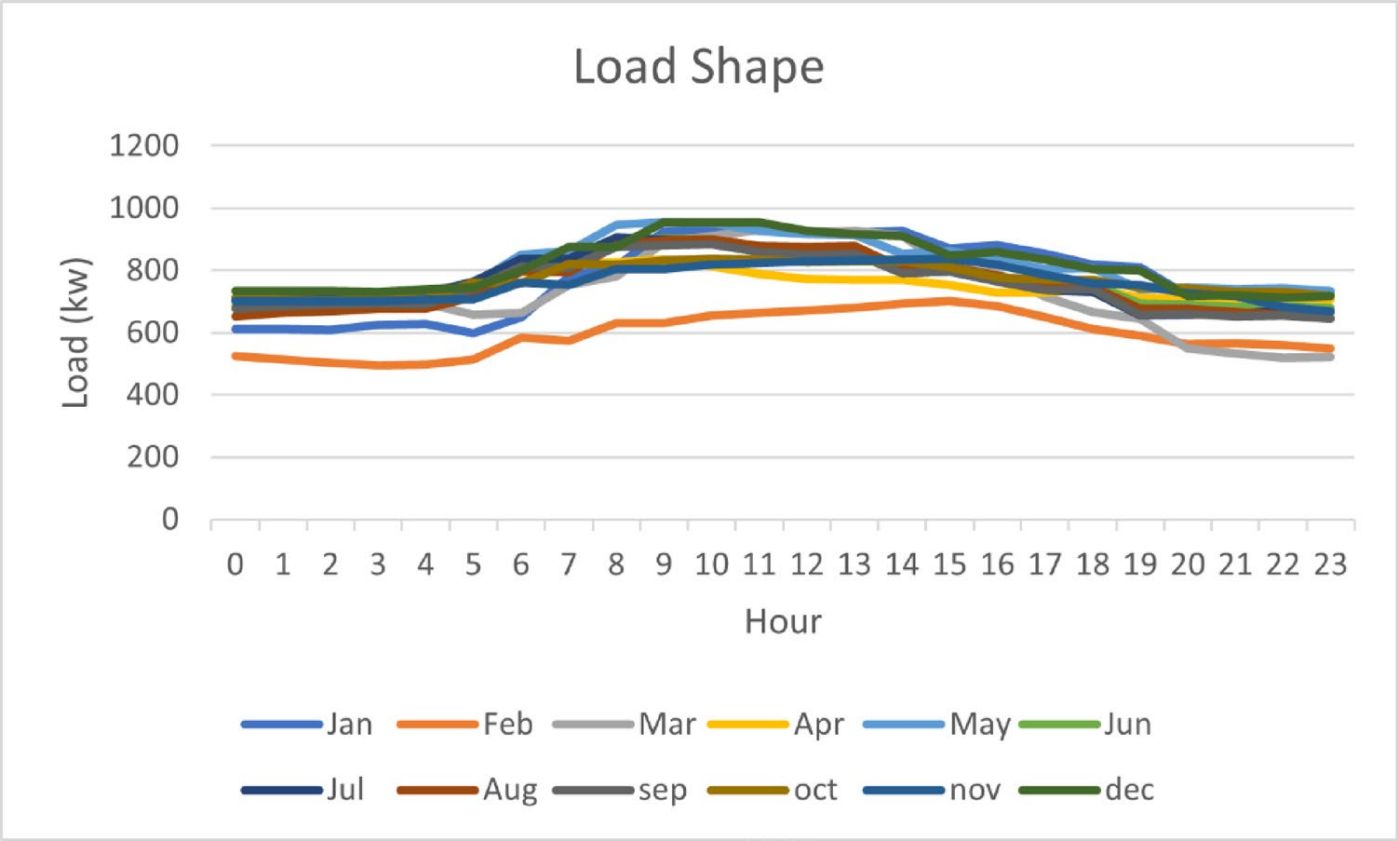
Electric load patterns.

Figure 2 shows the estimated monthly profile of the electrical load (kW) of the hospital during a full calendar year. There is a clear seasonal fluctuation in data, with the highest demand in the summer and the lowest load during spring and autumn.

## Simulation, results, and discussion

### Case study

**Fig. 3:**
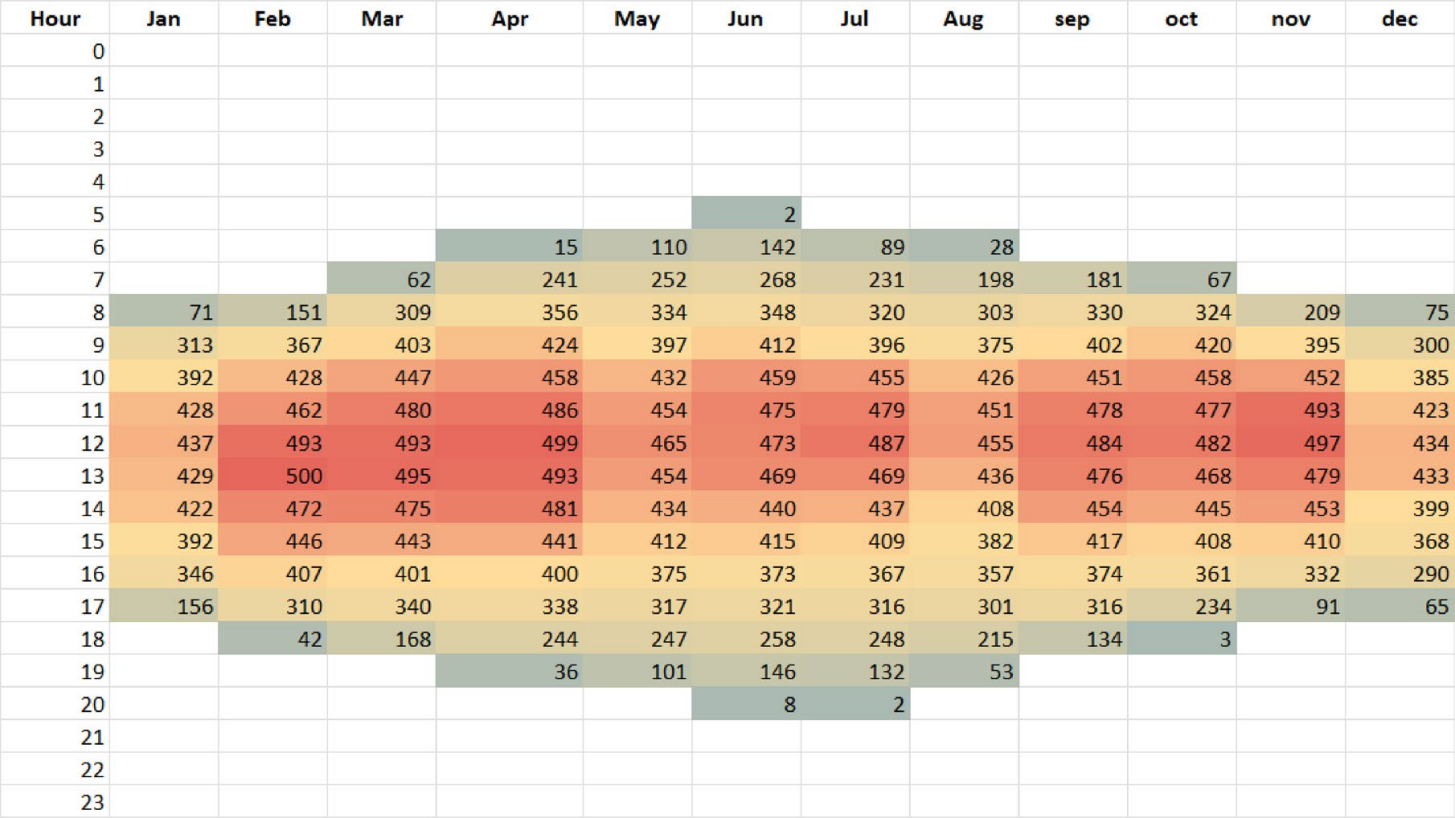
Global Horizontal Irradiance.

In the interest of this study, we have analyzed a hospital in Wilmington, DE, United States, which is subject to different climatic conditions^41^. Table 2 is a summary of the climatic conditions. Wilmington, DE, has a moderate solar irradiance, as shown in Fig. 3. The values in Table 2 are long-term annual averages.

Figure 2 presents the electrical load profile of the hospital, which forms the basic building block of the design and analysis of the microgrid. The electrical power that the facility consumes per year is a total of 7,300,001 kWh. The data outline clear and foreseeable trends of energy consumption during the day and season.

A distinguishable diurnal trend is formed, with a low base of demand in the nocturnal hours (around 00:00–06:00). The demand begins a strong upward trend around 07:00, which coincides with the beginning of the active working schedule of the hospital. The load maintains a plateau in the daytime, peaks at 11:00–16:00, and slowly decreases towards the evening.

The profile also indicates that there is a high amount of seasonal variation, with the greatest amount of energy consumption being recorded during the summer period (June-August) and, to a lesser extent, during winter. The summer peak demand, which is established mainly by cooling loads, reaches about 1150 kW. The months of transition (spring and autumn) have a rather low total demand. The dual-seasonality difference observed, along with the moderate solar irradiance typical of Wilmington, is a critical parameter in the analysis of the performance of the renewable energy sources on an annual basis, as well as in defining the optimum structure of the system.

All the input parameters used in the REopt model have been fully summarized in Appendix A, including detailed cost assumptions on each of the technologies, the utility rate structure, financial parameters (discount rate, analysis period, and escalation rates), and settings related to resilience scenarios. These parameters form the fundamental foundation on which all the economic calculations that have been incorporated in this study are built.

**Table 2:** Meteorological characteristics of the study location.

Site	Latitude	Longitude	$\:{\:\:\:\:\:\mathbf{m}\mathbf{e}\mathbf{a}\mathbf{n}\:\mathbf{G}\mathbf{H}\mathbf{I}}^{\boldsymbol{a}}$ (W/m²)	Wind speed at 50 m (m/s)	Min Temp (ºc)	AVG Temp (ºc)	Max Temperature (ºc)
Wilmington, Delaware	40º N	76º W	4.27	5.35	−18	12	34

a Global Horizontal Irradiance (W/m²).

### Results

#### Baseline scenario: financial optimization without resilience constraints

The microgrid was initially sized and dispatched based solely on economic optimization, without consideration of resilience metrics. This scenario establishes an optimal financial baseline against which subsequent resilience-oriented scenarios are compared.

A table of the optimization results was found in Table 3, where the largest photovoltaic array with the least Net Present Cost (NPC) was found to be 2,299 kWdc. This configuration would result in an NPC reduction (compared to the Business-as-Usual (BAU) scenario) of about 14% as the hospital relies solely on the utility grid. In addition to the financial savings, the PV integration inculcates a significant 26.67-percent cut in the CO_2_ emission, thus highlighting the environmental co-benefits that are embedded within the system. Note: The environmental benefit is mentioned here to reaffirm the all-embracing value proposition of the baseline, but it is not brought to the fore as a key concern in later resilience situations.

**Fig. 4:**
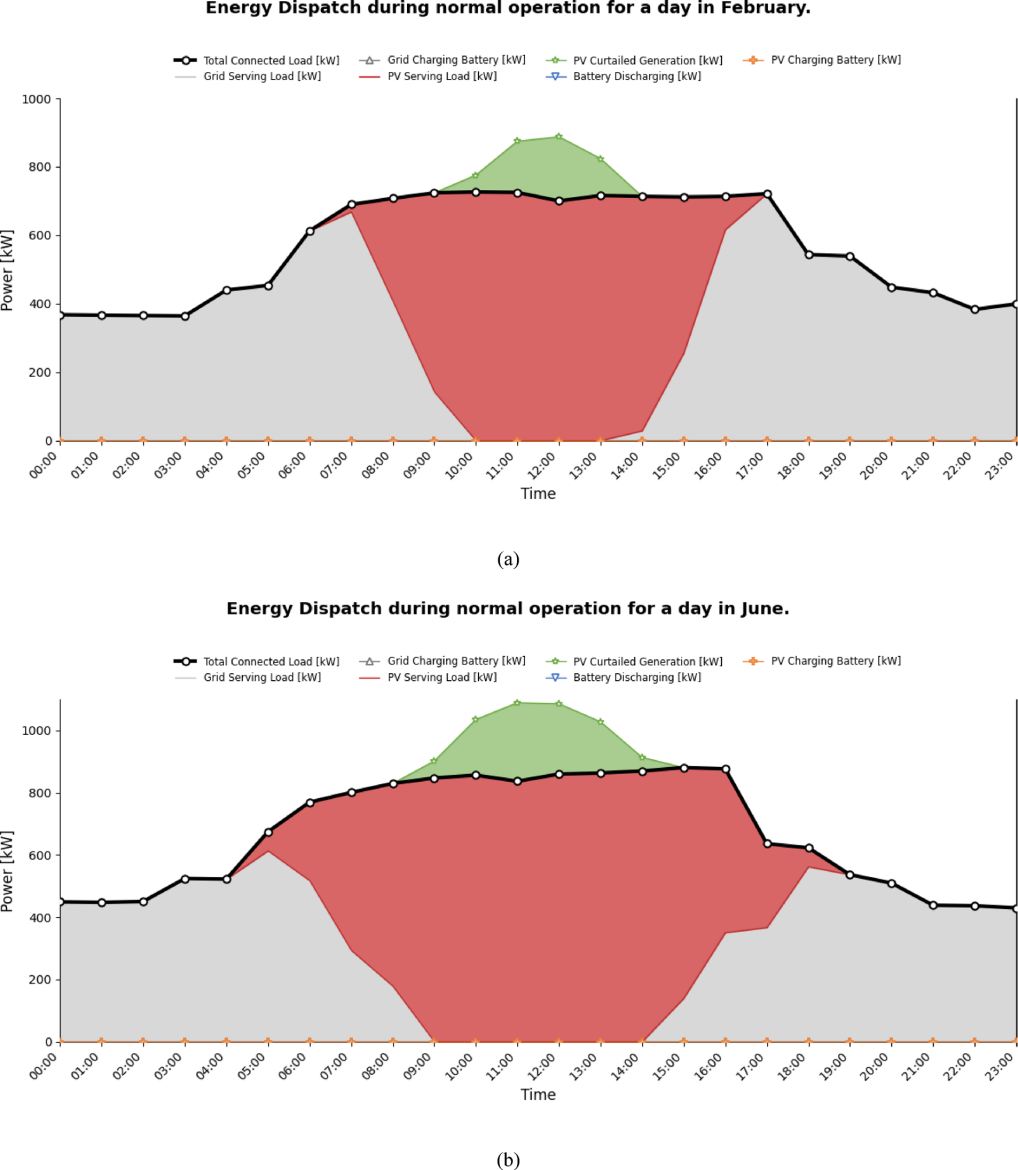
Energy dispatch for a day in June for the financial case. (**a**) February month (**b**) June month.

Optimal day-to-day energy dispatch strategy during a normal operation in February and June has been visualized in Fig. 4. The system gives more emphasis to photovoltaic production in order to meet the load of the hospital during the daytime. As Fig. 4a shows, the PV array can provide almost the full load in optimal production periods, and any excess production is curtailed as indicated in the PV curtailment profile. During the night or when the isolation is low, the remaining load is fed by the utility grid. A similar pattern of dispatch is observed in Fig. 4b, the dispatch pattern of June, though greater solar irradiance causes more PV generation, which will serve a larger proportion of the load and produce a larger portion of curtailed energy generation during the midday hours.

**Table 3:** Sizing and economic results for financial baseline scenario.

Case	PV (kW)	Total cycle cost	BAU	Savings	Savings %	Reduction in CO2 from BAU%
financial	2299	$12,082,884	$14,033,309	$1,950,425	14%	26.67%

This financial-only configuration serves as the key comparator for all subsequent resilience scenarios. In doing so, the total savings in resilience-constrained designs can be broken down into two parts: (1) the underlying savings of the PV-plus-battery design as compared to grid-only supply, and (2) the incremental cost or benefit associated with the resilience constraints. This is crucial for hospital administrators to determine if investing in resilience is a net cost or benefit for the hospital system or whether it is cross-subsidized by the renewable microgrid economics. Interestingly, the financial-only optimization failed to select a battery energy storage system because the economic case of storage under the current utility rate structure and PV costs did not justify the capital investment. Any surplus PV production in excess of the instantaneous load is, hence, curtailed or exported to the grid.

#### Resilience under various outage criteria

The resilience of the microgrid was evaluated with the help of a systematic analysis of the effectiveness of the microgrid under a set of premeditated outage events. To address the research question, the goal of this analytical investigation was to determine an optimal system setup to minimize the Net Present Cost (NPC) and, at the same time, maintain a predetermined level of critical load during instances of grid outage. The main variables that affect the operational strategy include the time the outage should be initiated, the particular calendar date, and the percentage of the total load that should be considered to be critical.

In order to test the functionality of the system during the seasonally extreme conditions, the investigation simulated four different outage events, each with a seven-hour duration. When these events started, they were all strategically timed to coincide with the seasonal zeniths of the critical load profile:

T1: January 5th at 1:00 PM (Winter).

- T2: May 31 st at 12:00 PM (Spring).
- T3: August 9th at 11:00 AM (Summer).
- T4: September 27th at 11:00 AM (Autumn).

To quantify the resilience performance, we use the System Resilience Index (SRI) defined as:

8 $$\:\mathrm{S}\mathrm{R}\mathrm{I}=\frac{{E}_{served}\text{}\text{}}{{E}_{demanded}}$$

Where $\:{E}_{served}$ is the total energy supplied to critical loads during the outage period, and $\:{E}_{demanded}$is the total energy demanded by critical loads over the outage period. An SRI of 1.0 indicates that 100% of the critical load was met with no unserved energy. values less than 1.0 would indicate some load curtailment during the outage. This is calculated for each outage scenario and critical load level in the analysis below.

##### Economic and sizing analysis

The economic and sizing results for systems designed to handle these outages at different critical load levels are summarized in Table 4. The optimized Net Present Cost (NPC) ranges from USD 12.41 million to USD 12.84 million, representing significant savings of 9% to 10.7% compared to the Business-as-Usual (BAU) case.

A key observation from Table 4 is the relative insensitivity of the PV capacity and total NPC to the critical load level. This suggests that the base system is sized primarily to achieve economic viability under normal operation, with the incremental cost of supporting higher critical loads during outages being managed by a more intensive use of the existing diesel generator and a re-optimized battery bank, rather than a massive oversizing of the PV array.

**Fig. 5:**
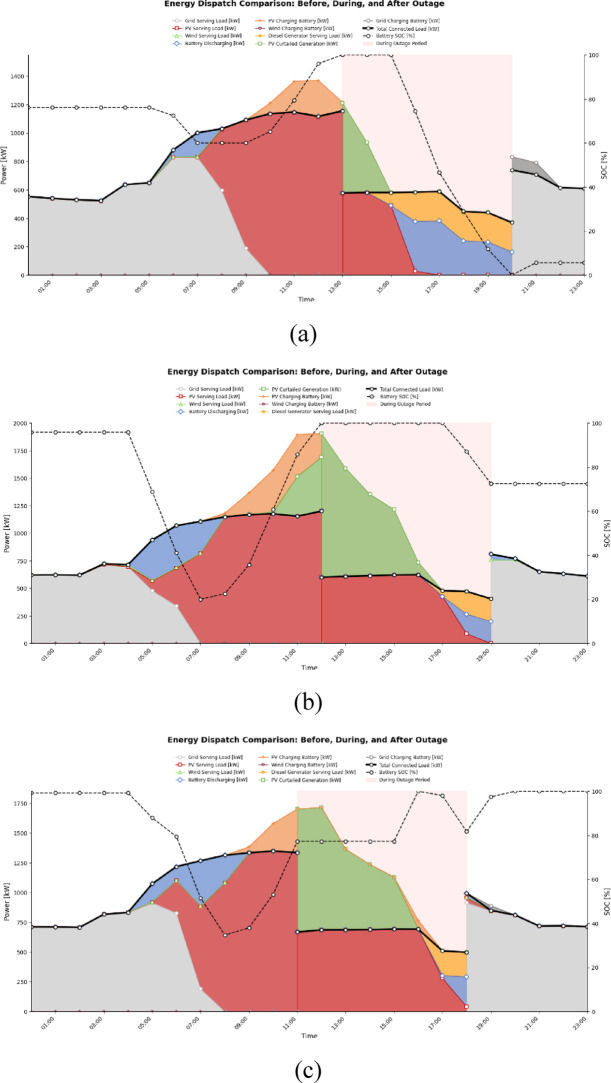

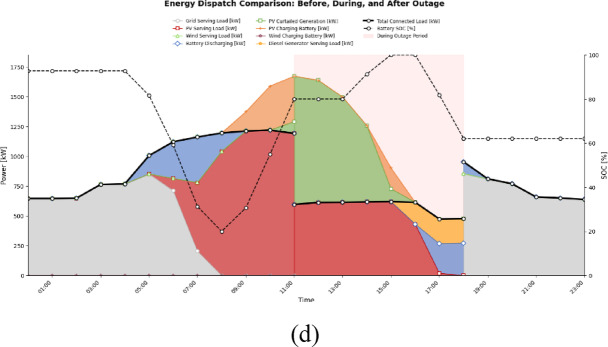
Power dispatch of the microgrid during a 7-hour outage under a 50% critical load. The sub-figures represent four seasonal outage scenarios: (**a**) January 5th at 1:00 PM, (**b**) May 31 st at 12:00 PM, (**c**) August 9th at 11:00 AM, and (**d**) September 27th at 11:00 AM.

**Table 4:** Economic and Sizing Results for Resilience Scenarios.

Parameter	50% Critical load	70% Critical load	100% critical load
System sizing			
PV (kW)	2,712	2,690	2,687
Batteries (kWh)	1,443	1,316	1,425
Diesel (kW)	207	425	768
Wind (kW)	24	38	68
Financial analysis			
BAU NPC (USD thousands)	$14,000	$14,000	$14,000
Optimized NPC (USD thousands)	$12,407	$12,574	$12,837
Savings vs. BAU (%)	10.7	10.2	9

Note: All scenarios are based on a 7-hour outage duration, tested across four seasonal start times (T1, T2, T3, T4).

**Fig. 6:**
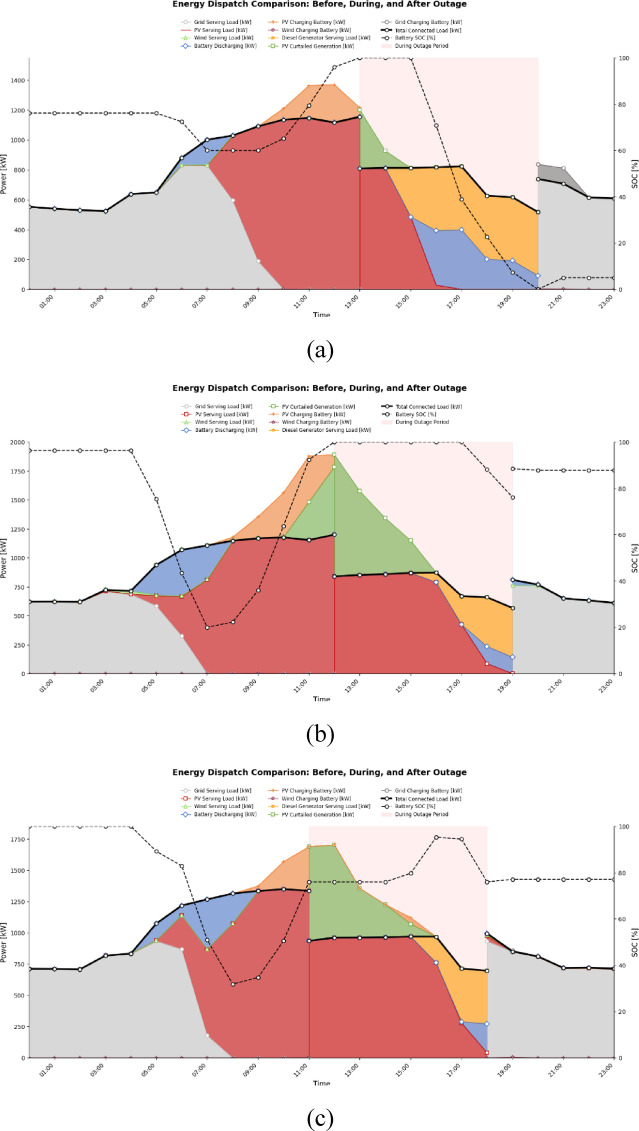

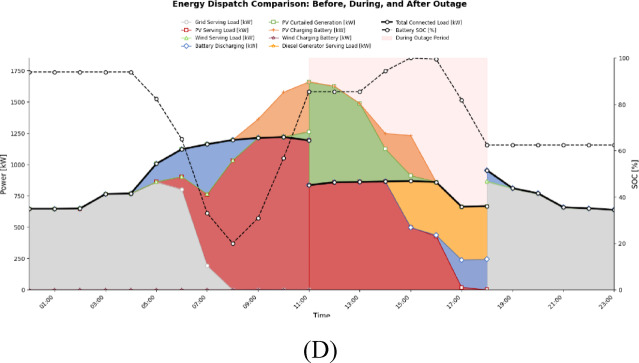
Power dispatch of the microgrid during a 7-hour outage under a 70% critical load. The sub-figures represent four seasonal outage scenarios: (**a**) January 5th at 1:00 PM, (**b**) May 31 st at 12:00 PM, (**c**) August 9th at 11:00 AM, and (**d**) September 27th at 11:00 AM.

##### Operational dispatch strategy

**Fig. 7:**
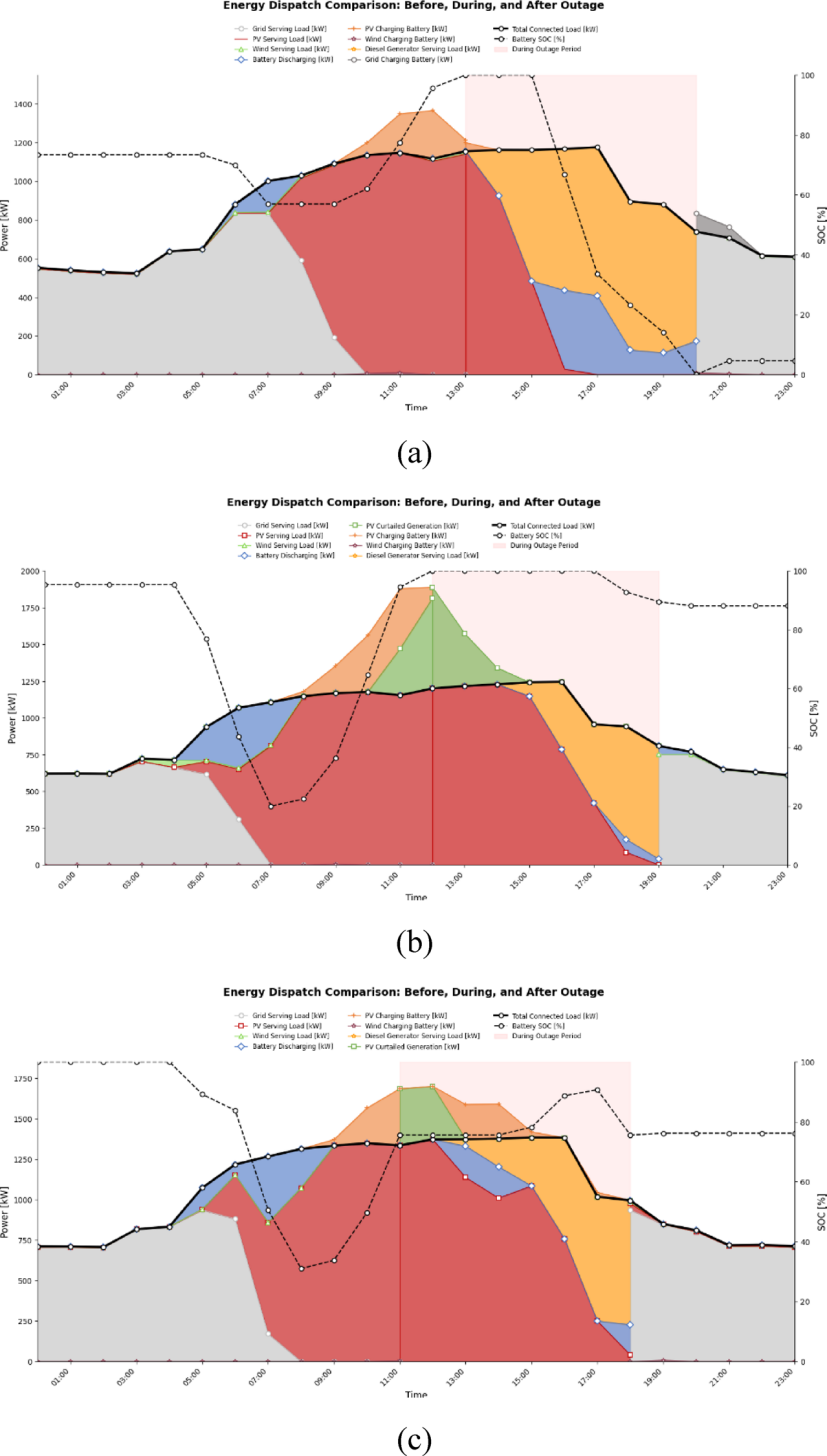

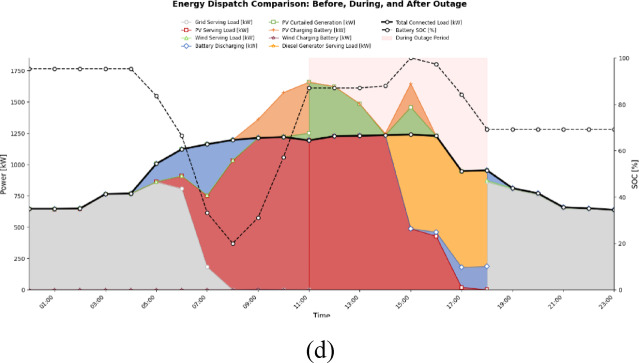
Power dispatch of the microgrid during a 7-hour outage under a 100% critical load. The sub-figures represent four seasonal outage scenarios: (**a**) January 5th at 1:00 PM, (b) May 31 st at 12:00 PM, (c) August 9th at 11:00 AM, and (d) September 27th at 11:00 AM.

The power dispatch strategy during a 7-hour outage is illustrated in Figs. 5 and 6, and Fig. 7, for 50%, 70%, and 100% critical load levels, respectively. During an outage, the microgrid operates in islanded mode, coordinating its assets to meet the critical load. For instance, in the winter outage (T1, Fig. 5a), the load is primarily supplied by the PV system and the diesel generator, with the Battery Energy Storage System (BESS) discharging to provide supplemental power.

The System Resilience Index (SRI) is 1.0 for all four seasonal 7-hour outage scenarios to confirm that the optimal microgrid design meets the 100% critical load demand without any unserved energy. This SRI score is also achieved at the 70% and 100% critical load levels, as the following dispatch analyses show.

For the 50% critical load scenario in Fig. 5, the system demonstrates efficient resource utilization across all seasons. In the winter outage (T1, Fig. 5a), the load is primarily supplied by the PV system and diesel generator, with the Battery Energy Storage System (BESS) providing supplemental power. The spring scenario (T2, Fig. 5b) shows optimal utilization of solar resources with minimal diesel support. Summer and autumn scenarios (T3 and T4, Fig. 5c and d) exhibit similar patterns with varying contributions from PV and battery resources based on seasonal solar availability.

For the 70% critical load scenario in Fig. 6, the operational strategy shifts to accommodate the higher power demand. The increased load level requires more substantial contributions from the diesel generator across all seasons, particularly during nighttime hours or periods of low solar generation. The battery system plays a more critical role in bridging gaps between renewable generation and load demand. In the scenario of spring (T2, Fig. 6b), the analysis maintains the most favorable terms, where the penetration of solar radiation is higher, in contrast to the conditions of winter (T1, Fig. 6a), which have to rely more on the diesel generator to maintain the high critical load.

In the case of 100% critical load (Fig. 7), which is the most severe resilience requirement, the system shows its full potential. The diesel generator takes the preeminent role in all situations, but the battery system plays a vital role in intermediate times. The summer scenario (T3, Fig. 7c) has the most balanced usage of resources due to the favorable conditions of the sun, and the winter scenario (T1, Fig. 7a) demands almost continuous running of the diesel to maintain the entire hospital workload. These adverse conditions notwithstanding, the microgrid ensures that there is no power outage by means of the coordinated dispatching of all available resources.

A distinctive feature of the resilience scenarios is the strategic deep discharge of the battery. Figures 5 and 6, and Fig. 7 show that the battery state of charge (SOC) is allowed to drop to 0% during grid outages. This practice needs to be justified because, if allowed to reach 0% while the battery is connected to the grid, the battery will degrade through processes such as anode copper dissolution and solid electrolyte interphase (SEI) instability.

The REopt model programs this deep-discharge protocol exclusively during grid outage events as a deliberate resilience strategy. The rationale is twofold. First, permitting full discharge maximizes the utilization of stored energy during emergencies, directly reducing reliance on the diesel generator and its associated fuel logistics. Second, by extracting maximum value from each kilowatt-hour of battery capacity during rare outage events, the model avoids the need for costly battery oversizing that would otherwise be required if a higher minimum SOC were enforced during outages. It is important to note that the lifecycle cost analysis accounts for a scheduled battery replacement at year 10 (see Appendix A), meaning that the incremental degradation from infrequent deep discharges is captured within the overall economic assessment. The cost savings from avoiding larger battery capacities outweigh the marginal degradation penalty from these emergency discharge events.

**Fig. 8:**
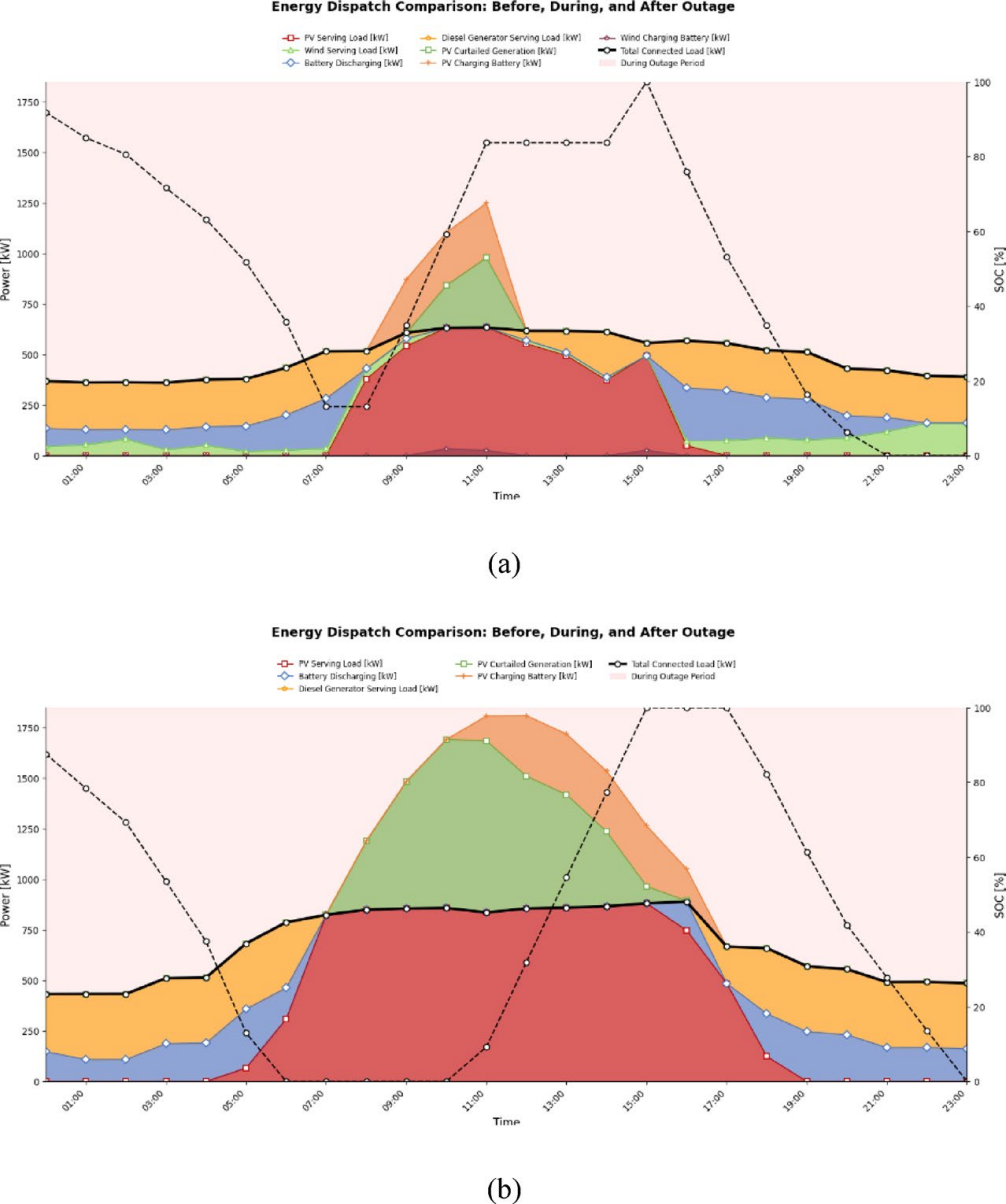
Power dispatch of the microgrid during a 24-hour outage under a 70% critical load. The sub-figures represent two seasonal outage scenarios: (**a**) January 7th at 00:00 AM, (**b**) July 07th at 00:00 AM.

A 24-hour outage scenario was simulated to assess the performance of the microgrid during long-duration grid failures, which are extreme events, in both a low-solar-resource month (January) and a high-solar-resource month (July) to hold the critical load level at 70%. An example of this operational dispatch case is also shown in Fig. 8, which shows how the system is available and the changing resource approach needed during a long period of islanded operation.

Figure 8a shows a sample of a power outage in a 24-hour outage that started in January. Under this low-solar condition, the system will be greatly dependent on the diesel generator to supply the baseload during the night and until the next day, when the solar resource is minimal and the battery is exhausted. The battery storage has a very important, but time-constrained, role in that it should be fully discharged during the first evening peak, followed by it starting to charge on the available PV generation on the second day. But this recharging is not always enough to offset the use of diesel. This situation highlights the point that during such long periods of outages occurring during seasons of low renewable resources, the diesel generator acts as a vital asset towards maintaining survivability.

As compared to this, the sample 24-hour outage in July (Fig. 8b) has a significantly different and more efficient dispatch. The excessive production caused by solar during the day supplies the critical load directly and charges the battery system completely. As a result, the microgrid will be able to run mainly on a mix of PV and battery storage during a considerable duration of the outage. The diesel generator is also minimized to just assisting in the evening and early morning hours when the state of charge of the battery is low. This shows that when the solar is favorable, the fuel usage and operational expenses can be significantly cut, even in the case of long-term emergencies.

A key finding from both sample scenarios is the critical importance of the battery’s daily cycling capability. Unlike in the 7-hour outages, where a single deep discharge sufficed, the battery in a 24-hour outage must effectively store daytime solar energy to cover the subsequent night, emphasizing the need for sufficient energy capacity (kWh) in addition to power rating (kW).

A 24-hour winter outage case (Fig. 8a, January, 70% critical load) provides a reference point to quantify on-site fuel storage demands- a critical logistical factor in the preparation of hospitals for emergency response. The diesel generator sent out 233 kWh of electrical power during this outage. This would translate to a minimum of 17.8 gallons (67.4 L) of diesel fuel being used up during the 24-hour outage period, based on the generator parameters listed in Appendix A (32.2% electrical efficiency and fuel higher heating value of 40.7 kWh per gallon).

Considering the above, the U.S. Federal Emergency Management Agency (FEMA) suggests that critical facilities should have at least 72 h of autonomous operation following a catastrophic event. To linearize the 24-hour consumption line, an outage of 72 h during similar conditions of low solar radiation would require around 53.4 gallons (202 L) of on-site diesel storage. This volumetric constraint points to a basic weakness of diesel-reliant backup schemes: the size of fuel storage is limited, and resupply chains are frequently disrupted during the same extreme weather conditions that cause grid outages. The strategic implication is obvious: a maximized photovoltaic and battery capacity will minimize the burden of fuel storage and the risk of operations in case of prolonged grid outages caused by fuel depletion. This observation supports the importance of the PV-plus-battery architecture as the primary resilience resource and the diesel generator as a supportive and not essential backup resource.

##### Sensitivity analysis of outage start time and duration

To investigate the robustness of the design, a sensitivity analysis was conducted on the outage start time and duration. Simulations were run for short-term (7-hour) and extended (24-hour) outages starting at midnight (T1), 8:00 AM (T2), and 4:00 PM (T3), as well as a full 24-hour outage starting at midnight (T4), for both January (low solar resource) and July (high solar resource) conditions.

The results, consolidated in Tables 3 and 4, reveal that systems designed for summer outages consistently achieve higher cost savings (up to 14.2%) than those for winter. The given phenomenon could be explained by the increased solar radiance throughout July, which leads to the reduction of reliance on the power supply based on diesel. In addition, the planned outages of 7 h, starting at 8:00 AM (time period T2), are often the most economical approach to take since their temporal distribution aligns with the solar peak production.

**Table 5:** Sensitivity analysis for january (low solar resource).

Jan												
Load level	50%				70%				100%			
Duration	Short			long	short			long	short			long
Starting time	T1	T2	T3	T4	T1	T2	T3	T4	T1	T2	T3	T4
PV	2,556	2,585	2,522	2,513	2,556	2,602	2,532	2,532	2580	2643	2597	2556
Battery kwh	1056	1432	291	1128	998	1480	1149	1149	1008	1170	1145	998
Wind	0	67	212	194	0	73	215	215	0	0	0	0
Diesel	164	0	103	119	277	134	233	233	445	376	533	554
BAU NPC (USD thousands)	14,000											
Optimized NPC (USD thousands)	12,127.605	12,064.133	12,127.947	12,129.927	12,198.122	12,155.670	12,214.965	12,215.535	12,308.854	12,299.808	12,370.139	12,371.935
NPC saving over BAU (USD thousands)	1872	1936	1872	1870	1802	1843	1785	1785	1691	1697	1629	1624
Savings vs. BAU (%)	13.37	13.83	13.37	13.36	12.87	13.17	12.75	12.75	12.08	12.14	11.64	11.6

**Table 6:** Sensitivity analysis for july (high solar resource).

Jul												
**Load level**	**50%**				**70%**				**100%**			
**Duration**	**short**			**long**	**short**			**long**	**short**			**long**
Starting time	T1	T2	T3	T4	T1	T2	T3	T4	T1	T2	T3	T4
PV	2620	2434	2799	2,625	2620	2611	2704	2,625	2,643	2737	2698	2633
Battery	1177	516	1948	1252	1177	774	1539	1,252	1151	1043	1475	1154
Wind	0	0	0	0	0	0	0	0	0	0	0	0
Diesel	174	0	0	169	329	1	237	324	564	0	486	572
BAU NPC (USD thousands)	14,000											
Optimized NPC (USD thousands)	12,152	12,012	12,138	12,040	12,250	12,026	12,236	12,250	12,404	12,054	12,376	12,410
NPC saving over BAU (USD thousands)	1848	1988	1862	1960	1750	1974	1764	1750	1596	1946	1624	1589
Savings vs. BAU (%)	13.2	14.2	13.3	14	12.5	14.1	12.6	12.5	11.4	13.9	11.6	11.3

Another interesting aspect of the sensitivity analysis in Tables 5 and 6 is that the small wind turbine capacities (67–215 kW) are only present in the low-solar-resource conditions in January. When the model is constrained to satisfy important loads during winter outages by only a modest amount of photovoltaic generation, it finds a marginal economic benefit in the diversification of the renewable portfolio: even a small measure of wind generation will eliminate the need to burn diesel fuel during multi-hour grid outages. But these optimized wind capacities are nothing compared to a facility of 7.3 GWh/year or less than 1% of the peak load, and wind capacity reduces to near zero in the July scenarios where there is plenty of solar resource. This finding should therefore be seen as a modeling fine-tuning at the optimal cost-function point and not a very solid design suggestion. To determine the feasibility of any material wind capacity in this location, a wind resource analysis specific to the project and including hub-height measurements would be necessary.

## Conclusions and future work

In this work, a techno-economic optimization model was used to design a grid-connected hospital microgrid considering resilience to grid interruptions of varying durations, timing, and critical load levels. The model was solved using NREL’s REopt tool to minimize the Net Present Cost (NPC) while ensuring the supply to critical loads. A medium-sized hospital was considered a case study with a microgrid consisting of solar PV, battery storage, and diesel generators.

Various outage scenarios (7-hour to 24-hour) were simulated to investigate the system’s capacity to deliver critical loads for four different initial seasons and three levels of critical loads (50%, 70%, and 100%). All of our resilient system configurations led to substantial Net Present Cost (NPC) savings (range: 9% to 14.2%) over the business-as-usual scenario (BAU) of 100% grid dependency. Importantly, these savings are not due to the resilience constraints but reflect the inherent economic advantages of the PV-plus-battery system compared to grid-only supply. When the resilient configurations are compared to the optimal financial-only system that was developed, which achieves a 14% NPC saving versus BAU, the incremental cost of resilience is 0.4% to 2.4% for the various scenarios. This result shows that the economic merits of the PV-plus-battery microgrid are so strong that resilience can be achieved at low incremental cost. The sensitivity analysis also shows that systems designed for summer outages always have the highest savings (14.2%) because of increased PV generation, but that systems designed for winter outages are also financially successful with savings of at least 11.6%, proving that the system is economical year-round.

The suggested process and results can be applied by hospital managers and energy managers who are considering including microgrids in their sustainability and resilience plans. Due to the sensitivity of microgrid sizing to the duration of outage, timing, season, and the degree of critical load, as demonstrated in this research paper, it is preferable that analysis should be performed based on site-specific load profiles and local tariff profiles.

Finally, the research potential can be extended in the future. A closer analysis of the workload in the hospital could be able to distinguish the life-saving medical equipment, the critical support services, and the non-essential loads and, therefore, a more refined tiered load management approach during outages. The techno-economic model can be added to a thorough environmental analysis that goes beyond the carbon dioxide to encompass the entire range of pollutants reported by the REopt platform, namely nitrogen oxides (NO_X_), sulfur dioxide (SO_2_), and fine particulate matter (PM_2.5_) using the Cambium grid emissions dataset of the PJM East region already imported into the current model configuration (see Appendix A). The direct and well-documented effects of these local air contaminants on respiratory health and cardiovascular health and their emission in the vicinity of a hospital, where patients with compromised health conditions are concentrated, are a particularly important externality to quantify across the outage scenarios considered in this study. Moreover, the economic potential of offering grid ancillary services with the battery storage system during normal operation to enhance the overall returns on investment may be investigated. Added to this would be a powerful step forward incorporating strong optimization to consider the uncertainties in outage prediction and renewable generation.

## Data Availability

All data generated or analyzed during this current study are included in this published article.

## Appendix A. Simulated model inputs and assumptions

**Table:**

Category	Parameter	Value/Setting
General & Site	Evaluation Name	hospital
	Site Location	Wilmington, DE (39.74, -75.55)
	Energy Goals	Cost-Savings, Resilience
	Technologies Selected	PV, Battery, Wind, Generator
	PV & Wind Space (Land)	1,000,000 acres
	Solver Optimality Tolerance	0.1%
Utility	Annual Energy Charge	$0.15/kWh
	Annual Demand Charge	$10.00/kW/month
Load Profile	Type	Simulated Building (Hospital)
	Annual Electric Consumption	7,300,001 kWh
Financial	Analysis Period	25 years
	Host Discount Rate (Nominal)^2^	6%
	Host Effective Tax Rate	26%
	Electricity Cost Escalation Rate (Nominal)	1.7%/year
	O&M Cost Escalation Rate	2.5%/year
	Generator Fuel Cost Escalation Rate (Nominal)	1.97%/year
Emissions	Cambium Location (Grid Region)	PJM East
	Cambium Levelization Years	25
Solar PV	Size Class	Industrial (2,001–10,000 kW)
	$1,600/kW-DC	$1,600/kW-DC
	O&M Cost	$16.0/kW-DC/year
	Array Type	Rooftop, Fixed
	Array Tilt	10.0 degrees
	Array Azimuth	180.0 degrees
	DC to AC Ratio	1.1
	System Losses	14%
	MACRS Schedule	7 years
	MACRS Bonus Depreciation	60%
Battery Storage	Energy Capacity Cost	$420.00/kWh
	Power Capacity Cost	$968.00/kWConstant Cost
	Constant Cost	$222,115
	Annual O&M (% of Capital)	2.5%
	Replacement Year	10
	Minimum State of Charge	20%
	Initial State of Charge	50%
	MACRS Schedule	7 years
	MACRS Bonus Depreciation	60%
Wind Turbine	Size Class	0:100
	Capital Cost	$4,760/kW
	O&M Cost	$42.0/kW/year
	MACRS Bonus Depreciation	60%
Emergency Generator (Diesel)	Capital Cost	$500.00/kW
	Fixed O&M Cost	$10.00/kW/year
	Diesel Fuel Cost	$2.25/gallon
	Electric Efficiency (Full Load)	32.2% (HHV)
	Electric Efficiency (Half Load)	32.2% (HHV)
	Fuel HHV	40.7 kWh/gallon
Resilience Scenario	Outage Start	July 7th, 12:00 AM
	24 h	24 h
	100% of Typical Load	100% of Typical Load

^2^This 6% nominal discount rate is a standard assumption on the economic analysis of energy projects in the United States, and is consistent with the default parameters used in NREL’s REopt model and the Annual Technology Baseline. To conduct hospital-specific feasibility studies, it is advisable to perform a sensitivity analysis of the discount rate, as healthcare facilities may offer lower discount rates because they are non-profit organizations.

## Abbreviations

BAU: Business as usual
BESS: Battery energy storage system
CRF: Capital recovery factor
DER: Distributed energy resources
DG: Diesel generator
ESS: Energy storage system
GHI: Global horizontal irradiance
LCOE: Levelized cost of energy
NPC: Net present cost
O&M: Operation and maintenance
PV: Photovoltaic
REopt: Renewable energy optimization tool
SOC: State of charge
V2G: Vehicle-to-grid
V2H: Vehicle-to-home
WT: Wind turbine
SRI: System resilience index

## Symbols

$$\:\mathrm{A}$$: Panel area ($$\:{m}^{2}$$)
$$\:{B}_{soc}$$: The state of charge of the battery
$$\:{\mathrm{C}}_{cap}$$: Capital cost ($)
$$\:{C}_{p}$$: Power coefficient for a wind turbine
$$\:A$$: Panel area ($$\:{m}^{2}$$)
$$\:{\mathrm{F}}_{0}$$: No-load fuel consumption ratio
$$\:{\mathrm{F}}_{1}$$: Marginal fuel consumption coefficient
$$\:\mathrm{G}$$: Solar irradiance
$$\:m$$: Project lifetime (years)
P: Electrical power output (kW)
$$\:{P}_{ch}\left(t\right)$$: The charging power at step t of time (kw)
$$\:{P}_{dis}\left(t\right)$$: The power that is discharging at time step t (kw)
$$\:{P}_{gen}$$: Electrical Power output of the DG (kW)
$$\:{P}_{pv}\left(t\right)$$: The power generated by photovoltaics in kilowatts
$$\:{P}_{wt}\left(t\right)$$: The wind turbine power output at time t (kw)
r: real interest rate (%)
t: Time (h)
V: The speed of wind (m/s)
Y: Equipment lifetime (years)
$$\:{\mathrm{Y}}_{gen}$$: Rated generator capacity (kW)

## Greek symbol

$\:{\eta\:}_{ch}$: Battery charging efficiency (%)
$\:{\eta\:}_{dis}$: Battery discharging efficiency (%)
$$\:{\eta\:}_{inv}$$: Inverter efficiency (%)
$\:{\eta\:}_{pv}$: PV system efficiency (%)
$\rho$: Density of air $(kg/m^3)$
